# Multi-Functional Macromers for Hydrogel Design in Biomedical Engineering and Regenerative Medicine

**DOI:** 10.3390/ijms161126056

**Published:** 2015-11-19

**Authors:** Michael C. Hacker, Hafiz Awais Nawaz

**Affiliations:** Institute of Pharmacy, Pharmaceutical Technology, Leipzig University, Eilenburger Str. 15a, D-04317 Leipzig, Germany; hn69sohi@studserv.uni-leipzig.de

**Keywords:** functional groups, chemical cross-linking, physical gelation, macromer, macromonomer, smart hydrogel, *N*-isopropylacrylamide, copolymer, click chemistry

## Abstract

Contemporary biomaterials are expected to provide tailored mechanical, biological and structural cues to encapsulated or invading cells in regenerative applications. In addition, the degradative properties of the material also have to be adjustable to the desired application. Oligo- or polymeric building blocks that can be further cross-linked into hydrogel networks, here addressed as macromers, appear as the prime option to assemble gels with the necessary degrees of freedom in the adjustment of the mentioned key parameters. Recent developments in the design of multi-functional macromers with two or more chemically different types of functionalities are summarized and discussed in this review illustrating recent trends in the development of advanced hydrogel building blocks for regenerative applications.

## 1. Introduction

Hydrogels have become essential tools in biomedical engineering and regenerative medicine [1,2,3]. Properties such as high water content as well as structural and mechanical similarity to natural extracellular matrix (ECM) have motivated the use of such systems for drug and gene delivery, cellular and tissue engineering as well as cell encapsulation and cell-based regenerative therapies [4,5,6]. A recent trend in biomaterial research is to impart specific properties, physico-chemical or biological in nature, to the material in order to maximize or tailor the regenerative potential of the biomaterial to a specific application [7]. Ideally, a smart or biomimetic material supports cultivation with cells that are relevant for the regeneration process and trigger cell differentiation towards a desired phenotype as warranted for concepts such as *in situ/in vivo* tissue engineering [8]. In order to mediate these specific properties, defined molecular functionalities or domains have to be incorporated in the hydrogel-forming polymers. With the objective to keep the hydrogel design flexible, to allow for an efficient degradability and to keep the fabrication/swelling process convenient, the use of low molecular weight polymeric building blocks is preferred over conventionally used high molecular weight, sometimes even branched polymers. Such building blocks also enable the fabrication of cell laden hydrogels by additive manufacturing technologies [9]. Established natural or synthetic hydrogel forming materials are often refined by the introduction of additional functional groups, for example to render the building blocks *smart* in the sense that the materials respond to precisely defined stimuli in a defined manner [7]. The type of stimuli that hydrogels have been made sensitive for, have become more diverse during the last decade. Progression from established concepts of temperature- and pH-sensitive materials has led to materials with stimuli-dependent shape memory effect [10], light dependent degradation [11] or other specific degradation behavior [4]. Such functionally refined materials hold promise for different industrial and biomedical applications [12], especially in controlled drug or gene delivery and for regenerative therapies.

This review focuses on current trends in the development of such oligomeric or polymeric building blocks for the design of cytocompatible hydrogels for regenerative applications. More precisely, we specifically focus on materials that contain at least two chemically different types of functional groups or properties, while at least one of these functionalities supports polymer network formation from these building blocks via physical or chemical cross-linking. The oligo- or polymeric building blocks considered here are addressed as macromers—a term, which is not clearly defined. The IUPAC Compendium of Chemical Terminology (IUPAC Gold Book) lists macromer as a possible short form of macromonomer, but at the same time strongly discourages this synonymous use of the terms. Macromonomers, on one hand, are defined as oligo- or polymeric molecules which each have one end-group that acts as a monomer molecule [13]. Consequently, each macromonomer contributes only a single monomer unit to a chain of the product graft polymer upon polymerization. The term macromer, on the other hand, is often used to describe oligo- or polymeric molecules that contain one or more reactive functional groups that act as a monomer molecule or that can form a chemical bond with a complementary chemical group, e.g., from another building block, under the formation of cross-linked networks. Here, we like to use the term macromer in this broader sense and provide a structured overview on such materials with oligofunctionality, which means that the macromer provides at least two different types of functional groups or domains. These functionalities include physically and chemically cross-linkable structures, functional groups for other conjugation chemistries as well as molecular structures that render a specific degradability to the macromer, mediate a specific biological property, such as integrin binding affinity that would promote cell adhesion, or impart shape memory properties. Analytical labels are not considered as specific functionalities in the context of this review.

## 2. Macromers with at Least Two Types of Functional Groups for Cross-Linking

Hydrogel mechanics is a key parameter with respect to regenerative applications as it affects the adherence of cells to the hydrogel, the migration of cells into the material and the differentiation pathway that a precursor cell will predominantly follow [14]. In the context of *in situ* tissue engineering, hydrogel mechanics is a critical material characteristic that can be used to instruct invading stem cells and has to be carefully adjusted to the specific application. Hydrogels are formed from hydrophilic macromolecules, which typically do not form mechanically strong intermolecular bonds due to a lack of strong disperse interactions. Cross-linking of macro(mono)mers, that can be formulated as solutions that are easy to process due to low viscosity, is a common strategy to obtain hydrogel materials with adjustable mechanical stability. One distinguishes between physical and chemical cross-linking reactions depending on the involved chemical functionalities [15,16,17,18]. Upon chemical cross-linking, covalent bonds are formed between at least one type of the hydrogel forming macromolecules. During physical cross-linking, strong, non-covalent interactions are formed between the constituent macromolecules. As physical cross-linking does not involve reactive moieties or chemical conversion of the involved molecules, such reactions have an inherent compatibility with live cells or sensitive proteins. In chemical cross-linking strategies, macromer reactivity and associated cytotoxicity have to be carefully balanced.

Physical gelation mechanisms are manifold. Typically, the gelation mechanism involves a thermodynamic alteration of the polymer solution that leads to an increase in polymer-polymer interactions at the expense of polymer-solvent (water) interactions. Such a gel formation can be triggered by even slight environmental changes and/or external stimuli, including light irradiation or a distinct change in solution pH, salt concentration and most prominently temperature [19,20]. Polymeric materials with these properties are generally addressed as stimuli-responsive macromolecules. Thermo-responsive materials are of particular interest when the transition temperature of the formulation can be adjusted to just below body temperature, which would make the formulation injectable in the sense that the formulation represents a viscous sol at room temperature and turns into an elastic gel when heated to 37 °C. Typically, this phase transition is caused by a coil-globe transition of the thermo-responsive macromolecule and occurs at a characteristic temperature, the critical solution temperature. Polymers that exhibit a phase separation on heating possess a lower critical solution temperature (LCST) while gelation upon cooling would be characterized by an upper critical solution temperature (UCST) [21]. Thermally induced phase transition occurs in solutions of macromolecules that have a balanced distribution of hydrophilic and hydrophobic structures in the molecule [22]. In aqueous solution, the molecules interact with water via their hydrophilic structures, which generates a certain order in the solution due to repulsion between solvent molecules and hydrophobic domains of the molecules. This entropic component becomes increasingly critical with increasing temperature until, above a critical solution temperature, partial desolvation of the macromers leads to gel formation [23].

Other physical cross-linking strategies involve hydrophobic interactions, hydrogen bonding, ionic interactions and fairly special mechanisms such as guest-host inclusion complexation and stereo-complexation [24,25].

**Figure 1:**
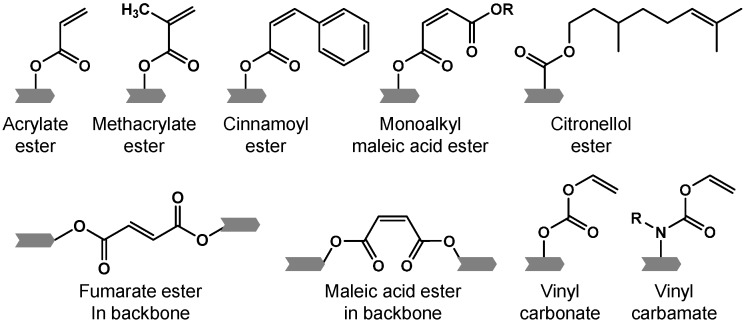
Selected functional groups used for chemical cross-linking of macromers by cross-polymerization. The symbol 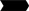 represents an oligomer segment that contains a functional group.

**Figure 2:**
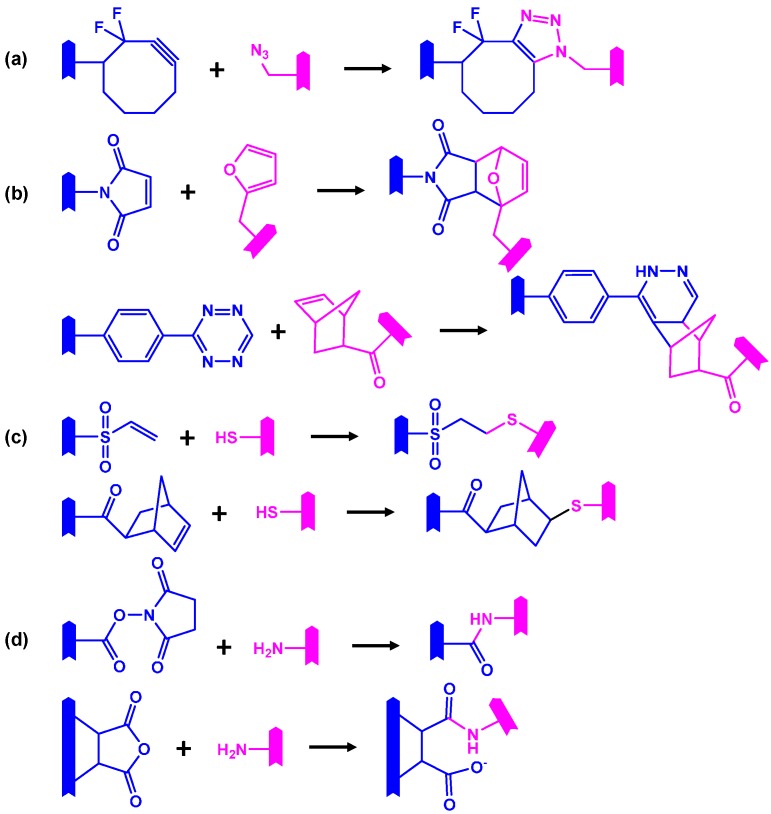
Selected conjugation reactions used for chemical cross-linking of macromers with corresponding functional groups (blue and magenta). The symbol 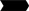 represents an oligomer segment that contains a functional group. (**a**) Strain-promoted metal-free cycloaddition of a difluorocyclooctyne with an azide; (**b**) Diels-Alder cycloaddition of a maleimide with a furane and below an Inverse electron demand Diels–Alder reaction of a tetrazine with a norbornene; (**c**) Thiol-ene reaction of a vinyl sulfone with a thiol through a Michael addition mechanism and below a free-radical thiol-ene addition of a thiol to a norbornene; (**d**) Amide formation of a primary amine with a succinimide ester or a cyclic anhydride (below).

Chemical gelation occurs when reactive moieties in macromers form covalent bonds in a chemical reaction between neighboring macromer molecules. Such chemical cross-links affect the swelling degree of the molecules and result in confined swelling ratios [26]. Although chemical gelation leads to networks with high mechanical stability and durability, such formulations might be associated with toxic effects of initiator, cross-linker or unwanted by-products [27]. There are two general classes of reactive moieties that can be used for chemical cross-linking of macromers: (1) functional groups that act as monomers in a polymerization reaction or (2) chemical moieties that take part in a conjugation reaction with a distinct complementary functional group. Examples of functional groups that are polymerized upon macromer cross-linking or cross-polymerization, to be more precise, include pendant acrylate, methacrylate, cinnamate, maleate or fumarate groups [28] (Figure 1). With regard to functional group toxicity and biocompatibility of degradation products, vinyl ester, vinyl carbonate and vinyl carbamate moieties have recently been established as potential alternatives [29]. Cross-polymerization of functional groups of class (1) is typically initiated in a radical mechanism either by UV light irradiation in the presence of a photoinitiator or by a temperature increase in combination with a thermoinitiator. The same radical cross-polymerization mechanism is applied when fumarate or maleate groups are incorporated in the repeating units of macromer chains [30,31,32]. Examples of pendant functionalities that can take part in conjugation reactions for chemical macromer cross-linking are summarized in Figure 2. Such conjugation reactions include azide-alkyne Huisgen cycloadditions, Diels-Alder cycloaddition, Thiol-ene and Michael addition chemistries [33], and amide formation [24,34] (Figure 2).

### 2.1. Macromers with Dual (Physical and Chemical) Gelation Properties

This review focuses on hydrogel-forming macromers with more than one type of chemical functionality per molecule. The most common combination that has been established thus far is the combination of functional groups for chemical and physical gelation within a macromer molecule. Formulations of such macromers undergo a so called tandem or dual gelation upon hydrogel formation during which covalent and non-covalent associations are established [35]. Hydrogels showing only physical or chemical cross-links are considered less advantageous [36]. Physically gelled hydrogels express gentle flow under minor stress while chemically gelled macromers result in higher degrees of swelling, which is considered disadvantageous for applications in confined volumes [36]. Tandem gelation mechanisms allow for better control and adjustment of key characteristics such as mechanical strength, swelling and flow properties.

In the design of chemically and physically gellable macromers one can consider different combinations of the above-described variants of both gelation mechanisms. Thermally induced gel formation of thermo-responsive macromers is the most commonly followed physical gelation strategy to date. Chemical structures that show thermo-responsive behavior include (1) homo- and copolymers of *N*-isopropyl acrylamide (NiPAAm); (2) block copolymers which contain hydrophilic poly(ethylene glycol) (PEG) blocks in combination with hydrophobic blocks of poly(propylene glycol) (PPG), biodegradable polylactide (PLA) or poly(propylene fumarate) (PPF), for example; and (3) poly(organo phosphazenes) (POP) [37,38,39,40].

#### 2.1.1. NiPAAm-Based Dual-Gelling Macromers

A variety of NiPAAm-based macromers has been described. Dual gelation strategies have been realized through the integration of different chemically cross-linkable moieties. NiPAAm-containing macromers are synthesized by polymerization of the acrylamide monomer NiPAAm. The underlying polymerization mechanism, living or free radical, allows for a fairly straight-forward integration of other (meth)acrylate, (meth)acrylamide or even vinyl comonomers with additional functional groups [41]. The Vernon group has synthesized NiPAAm-based macromers with *N*-acryloxysuccinimide as comonomer, which introduced reactive esters to the macromer, that could be further modified with cysteamine to obtain a thiol-group containing poly(NiPAAm*-co-*cysteamine)-based macromer [42]. Such pendant thiol groups can be utilized for bioconjugation reactions or chemical cross-linking reactions, e.g., in a Michael addition-type reaction with acrylate [36], acrylamide [43] or vinyl sulfone groups [44], or in a photo-initiated thiol-ene “click” reaction [18,45] (Figure 2). Accordingly, NiPAAm-based copolymers with pendant acrylate and vinylsulfone groups were synthesized as reaction partners for the formulation of dual gelling hydrogels [36]. Michael addition reactions generally occur between nucleophiles (amines, thiols) and olefinic double bonds at a fast reaction rate with high efficiency at physiological pH and temperature, which is of great interest for injectable formulations and direct cell encapsulation [46]. Macromers with vinyl groups, as compared to more polar acrylate groups, have shown a faster reaction with thiol groups [36,42]. The term thiol-ene reaction summarizes a wider array of reactions between a thiol and an unsaturated carbon-carbon bond [47,48]. A Michael addition reaction that involves thiol groups is considered a specific sub-type of a thiol-ene reaction [49]. While the Michael addition of a thiol to a polarized C=C double bond is a base/nucleophile-initiated thiol-ene reaction, there are also radical-mediated hydrothiolation reaction of a C=C bond that can be performed on less reactive double bonds. Later in this review, we will present examples of photo-initiated thiol-ene reactions involving cysteine thiol groups and norbornene-modified macromers [45].

Another example of chemical functionalities that have been introduced into NiPAAm-based macromers for chemical cross-linking are ketone and aldehyde groups, that might need to be protected in the corresponding monomers upon macromer chain polymerization [50]. The electrophilic groups were cross-linked with a hydrazide-functionalized NiPAAm-based macromer [51]. The gelation time, degradation kinetics and hydrogel mechanics could be independently controlled by tuning the ratio of aldehyde to ketone functional groups as well as the total number of ketone groups in the electrophilic macromers. The ketone-functionalized macromers exhibited improved cytocompatibility relative to macromers with pendant aldehyde groups.

**Figure 3:**
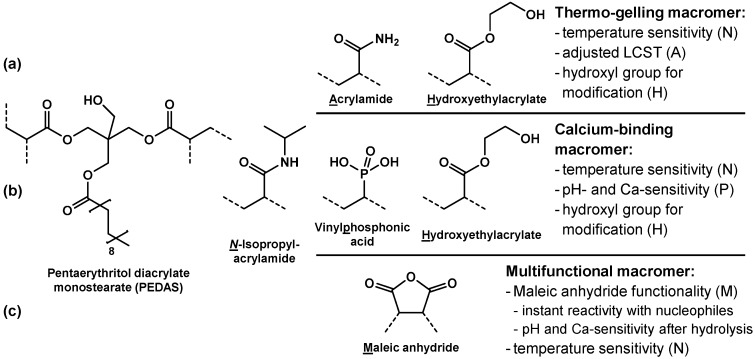
Amphiphilic pentaerythritol diacrylate monostearate (PEDAS)- and NiPAAm-based macromers with a list of key properties imparted by the constituent comonomers abbreviated by the underlined character in the macromer name. Dotted lines illustrate the polymeric backbone of the macromer. (**a**) Thermogelling macromer that was rendered chemically gellable after methacrylation of H [52]; (**b**) Calcium-binding macromer with vinylphosphonic acid as comonomer [55]; (**c**) Multifunctional maleic anhydride-containing macromer [56].

Hacker *et al.* [52] synthesized a series of amphiphilic NiPAAm-based macromers with dual gelation properties (Figure 3a). The macromers consisted of NiPAAm and a low content of lipophilic pentaerythritol diacrylate monostearate (PEDAS) for hydrophobic interactions within macromers and with proteins and cells. The LCST of the macromers was adjusted with hydrophilic acrylamide and 2-hydroxyethyl acrylate as further comonomers. The latter provided pendant hydroxyl groups that could be modified by (meth)acrylation, which yields chemically cross-linkable, thermogelling, and potentially biodegradable macromers. Cytotoxicity investigations revealed that hydrogel formation and cross-linking within a time frame of up to 6 h would allow for viable cell encapsulation [53]. Mesenchymal stem cells were encapsulated in the hydrogels and remained viable over a period of three weeks *in vitro* [54]. Osteogenic differentiation markers were detected when the cells were cultured with osteogenic supplements and plain gels mineralized under these conditions. Calcium-ion sensitive macromers were developed utilizing the same general amphiphilic design through the incorporation of vinylphosphonic acid as comonomer [55] (Figure 3b). Recently, we further extended this macromer platform by the incorporation of maleic anhydride as comonomer, which yielded thermo-sensitive macromers with reactive anhydride groups along the oligomer chain [56] (Figure 3c). The anhydride groups showed high reactivity against amines as illustrated in cross-linking reactions with polymeric di- and trivalent amines. Upon chemical cross-linking, an amide bond is formed between the anhydride and a primary amine. In addition, a carboxylate group is generated which increases the hydrophilicity of the macromer. Anhydrides that are not involved in an amide formation reaction quickly hydrolyze in physiological environments and macromer reactivity but also potential toxicity of these moieties is lost. Water-soluble macromers showed thermally induced phase transitions and the transition temperature could be modulated by solution pH and calcium ion concentration. The maleic anhydride-containing macromers were effective cross-linkers in the fabrication of gelatin hydrogels and gelatin microparticles [57]. Macromer composition and concentration as well as gelatin-to-macromer-ratio were identified as parameters for the adjustment of hydrogel properties. Hydrogels with good cytocompatibility and elastic moduli between 1 and 10 kPa could be fabricated. With regard to the fabrication of cross-linked gelatin microparticles, the macromers yielded cross-linking degrees matching those of glutaraldehyde with less unwanted structural modification and the opportunity for additional chemical modifications.

**Figure 4:**
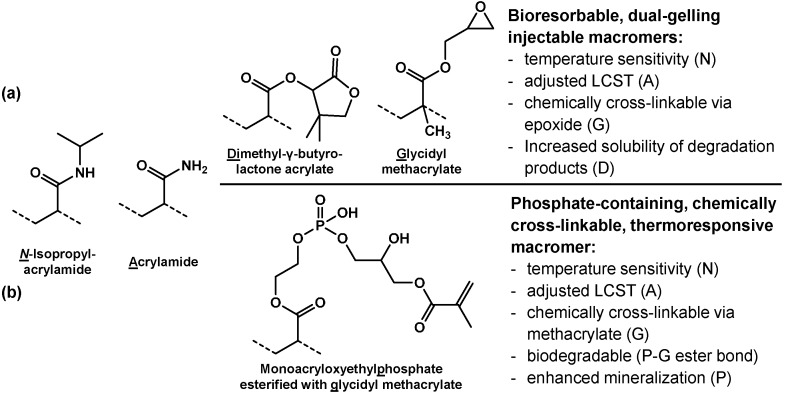
Linear NiPAAm/acrylamide-based macromers with a list of key properties imparted by the constituent comonomers abbreviated by the underlined character of the macromer name. Dotted lines illustrate the polymeric backbone of the macromer. (**a**) Dual-gelling macromer with pendant degradable functionalities (D) [60]; (**b**) Phosphate-containing degradable macromer [62].

The Mikos group has also progressed the concept of NiPAAm-based hydrogel forming macromers with at least two functionalities. First, macromers were synthesized by copolymerization of NiPAAm with glycidyl methacrylate that showed dual gelation properties in mixtures with polyamidoamine-based diamines, which served as reaction partners during the chemical cross-linking reaction of the macromer epoxy groups with amines of the polyamidoamines [58]. Initial thermogellation occurred within a few seconds and subsequent chemical gelation was completed in less than 3 h and classified as rapid and facile. The water-soluble and degradable polyamidoamine diamine components of the hydrogels offer high design flexibility and hydrophilicity, degree of hydrogel swelling or syneresis, degradation time scale, and degree of cross-linking can be tuned [59]. Upon degradation of this hydrogel formulation, the polymeric NiPAAm containing fragments have limited solubility in physiological media because the macromer structure has not been significantly altered and does not interact with water under these conditions—a characteristic that was initially intended to achieve physical gelation and is responsible for postformation hydrogel syneresis. The solubility of the degradation products can be effectively increased through the incorporation of charged groups that form after the gelation process has been completed and the specific phase transition properties are not required any more. In a further improved hydrogel system, the formation of charged hydrophilic moieties was realized by the integration of hydrolyzable lactone rings via dimethyl-γ-butyrolactone acrylate as comonomer [60] (Figure 4a). In order to adjust the LCST of the macromers, hydrophilic acrylamide was incorporated as fourth comonomer. The hydrogels allowed for the encapsulation of viable mesenchymal stem cells (MSCs) and were evaluated for cell delivery to a cranial defect [61].

Another dual-gelling macromer was synthesized from NiPAAm, acrylamide and monoacryloxyethyl phosphate [62] (Figure 4b). The macromers were then rendered chemically gellable by functionalization of pendant phosphate groups with glycidyl methacrylate via degradable phosphate ester bonds. The macromers could be tuned to undergo phase transition between room temperature and physiologic temperature. Chemical cross-linking stabilized the gels and mitigated syneresis at physiological temperature. The resulting gels were shown to degrade into soluble fragments and exhibited good cytocompatibility. The phosphate ester bonds in the hydrogels were sensitive to alkaline phosphatase, which provides an on-demand enzymatic degradation mechanism as this enzyme is secreted in osteogenically differentiating cells. These properties motivated an application in bone tissue engineering, and MSCs were encapsulated with high viability and differentiated towards the osteogenic lineage during *in vitro* cultivation over 28 days [63]. Cell-laden hydrogels were shown to undergo significant mineralization *in vitro* and could be placed into cranial defects in rats, where they underwent significant degradation and improved bone bridging of the defect.

Several conjugation strategies have been implemented in macromers to achieve effective chemical cross-linking and common conjugation chemistries have been exemplified above (Figure 2). Activated esters, especially succinimide esters, are common reactive derivatives of carboxylates with the ability to form amides with primary amine groups of another molecule under physiological conditions without further activation. This strategy has been employed to form cross-linked injectable hydrogels with thermosensitivity composed of type I collagen, which comprises primary amine groups, and a NiPAAm-containing macromer comprising reactive succinimide esters: poly(NiPAAm-*co*-polylactide-hydroxyethyl methacrylate(HEMAPLA)-*co*-acrylic acid(AAc)-*co*-*N*-acryloxysuccinimide (NAS)) [64]. The comonomer HEMAPLA introduced biodegradability by increasing backbone solubility upon PLA hydrolysis. AAc served to manipulate polymer hydrophilicity and NAS provided the activated esters for conjugation of bioactive molecules, such as type I collagen in this specific case.

With the rise of effective conjugation reactions, so called “click” reactions, between small molecules that react in a selective, fast and efficient manner several attempts have been made to utilize these chemistries for the chemical cross-linking of hydrogel-forming macromers. One example of these chemistries is the Diels-Alder (DA) cycloaddition reaction between electron-rich dienes and electron-poor dienophiles (Figure 2). This reaction mechanism is characterized by a high selectivity, the absence of a catalyst and side products and compatibility with aqueous environments [27]. A typical DA reaction is reversible, for example through the application of heat energy. The Diels-Alder mechanism with inverse electron demand (IEDDA), in contrast, is an irreversible cycloaddition between an electron-rich dienophile and an electron-poor diene, e.g., tetrazines, that was found to be a valuable tool for effective bioorthogonal conjugations [65,66,67]. Macromers that combined thermogelling properties and functional groups for cross-linking by a DA mechanism are poly(*N*,*N*-dimethylacrylamide-*co*-furfuryl methacrylate) and poly(NiPAAm-*co*-furfuryl methacrylate) [68,69]. The furfuryl groups act as dienes and react with maleimide groups (dienophile) of the bifunctional PEG-based cross-linkers *N*-[4-(formyl polyethylene glycol ester) bismaleimide or *N*-maleoyl-l-leucine polyethylene glycol ester bismaleimide. Gelation rate of these formulations could be tuned and cross-linking was thermally reversible. NiPAAm-containing macromers with functionalities for azide-alkyne Huisgen cycloaddition reactions have also been described [70]. Both alkyne- and azide-modified macromers have been synthesized by post-polymerization derivatization of poly(NiPAAm-*co*-HEMA). Thermoresponsive hydrogels could be cross-linked from solution of both macromers in the presence of catalytic amounts of Cu(I). Cellulose- and β-cyclodextrin-containing thermosensitive hydrogels were prepared from either azide-modified cellulose and alkyne-modified poly(NiPAAm-*co*-HEMA) [71] or alkyne-modified β*-*cyclodextrin and azide-modified poly(NiPAAm-*co*-HEMA) [72].

**Figure 5:**
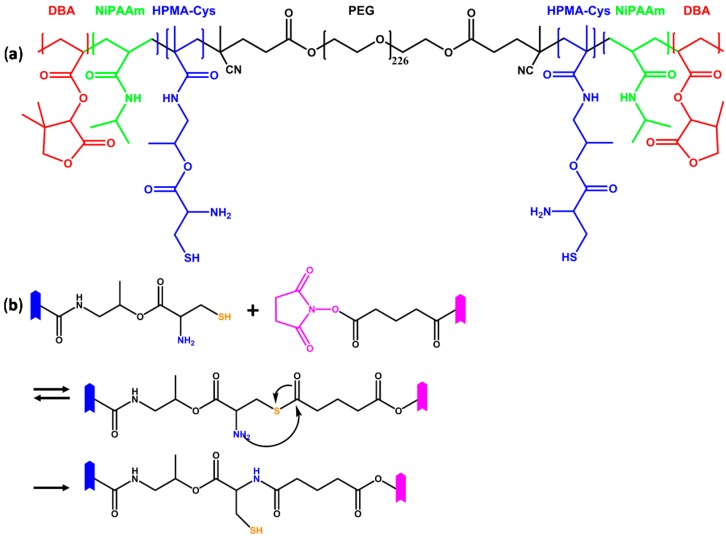
(**a**) Thermoresponsive macromer that can be chemically cross-linked by oxo-ester mediated native chemical ligation composed of a poly(ethylene glycol) (PEG) macroinitiator, thermosensitive NiPAAm (green), degradable dimethyl-γ-butyrolactone acrylate (DBA, red) and *N*-(2-hydroxypropyl)-methacrylamide-cysteine (HPMA-Cys, blue) bearing thiol groups [74]; (**b**) Mechanism of oxo-ester mediated native chemical ligation between thiol group (orange) of the macromer and an NHS-ester-functionalized cross-linker (magenta) [77]. The symbol 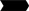 represents an oligomer segment that contains a functional group (blue, magenta). The initial reaction is a reversible transthioesterification step which is chemoselective and regioselective. The thioester intermediate rearranges by an intramolecular *S*,*N*-acyl shift that results in the formation of a stable native amide. Note that the resulting network provides fee thiol moieties for additional functionalization reactions.

Another recently used chemoselective reaction used for the cross-linking of thermoresponsive macromers is native chemical ligation (NCL) [73,74]. This chemistry involves the reaction between an *N*-terminal cysteine moiety and a thioester group through a reversible transthioesterification and terminates with an irreversible amide formation [75]. The reaction has advantages as it operates without catalyst at mild conditions and yields hydrogel that can be further functionalized [76]. However, it releases ethyl thioglycolate, a thiol byproduct, which has recently been shown to be cytotoxic. As an alternative, oxo-ester mediated native chemical ligation (OMNCL) has been presented that avoids toxic byproduct production and showed high chemoselectivity and cytocompatibility [77]. Recently, Boere *et al.* [74] reported on the synthesis of multifunctional macromer and hydrogel utilizing the OMNCL technique. An ABA macromer was synthesized in which the A block was synthesized by free radical polymerization and comprised of NiPAAm (to provide thermosensitivity), dimethyl-γ-butyrolactone acrylate (DBA, to provide controllable degradation) and *N*-(2-hydroxypropyl)-methacrylamide-cysteine (HPMA-Cys, to provide thiol groups for chemoselective ligation) and the B block was a hydrophilic PEG-di-4,4′-azobis(4-cyanopentanoic acid) ester macroinitiator (Figure 5a). The hydrogel was formed upon cross-linking the macromer with functionalized PEG molecule, e.g., 8-arm PEG-NHS ester (Figure 5b).

#### 2.1.2. Copolymer-Based Dual-Gelling Macromers

Amphiphilic block copolymers can be designed to form aqueous solutions that show lower critical phase separation below body temperature and can thus be used as injectable hydrogel formulations. Poloxamers, a group of non-degradable triblock copolymers of PEG and poly(propylene glycol) (PPG), are common pharmaceutical excipients and an example of such materials. Dual-gelling poloxamer-based macromers, which yield hydrogels with improved stability and residence time for drug delivery and tissue engineering applications, have been synthesized by end functionalization of the block copolymer with cross-polymerizable acrylate or methacrylate moieties [78,79]. Conversion of the hydroxyl endgroups to methacryloyl-depsipeptides resulted in materials with a controllable degradation rate dependent on the building blocks of the depsipeptide units [80]. Similar macromers have been used for tissue printing applications, and MSCs showed improved viability and osteogenic differentiation as compared to unphotopolymerized control hydrogels [81].

Another chemical cross-linking chemistry was used in a silane end-capped poloxamer macromer [82]. The macromer retained the thermo-gelling characteristics of the original poloxamer. Over time, the ethoxysilane groups hydrolyzed and the resulting silanol moieties chemically cross-linked in a condensation reaction. The hydrogels exhibited gradually increasing mechanical stability and compressive moduli.

An early example of dual-gelling macromers employed poloxamer-type macromers and Michael addition conjugation chemistry [83,84]. Specifically, the terminal hydroxyl groups of four-armed poloxamer-type block polyethers (Tetronic, BASF Corporation, Florham Park, NJ, USA) were converted to thioacetic esters via a vinyl ether intermediate. The pendent thioacetic ester groups produced thiols after basic treatment and served as donor in the conjugation reaction. An acrylate-bearing Tetronic was synthesized as acceptor for the addition. The network properties of the resulting hydrogels were determined by molecular weight and degree of functionalization of the Tetronic precursor and were stable for a few weeks. The use of a different Michael acceptor, such as a Tetronic tetravinyl sulfone, was considered to yield more stable hydrogels.

As poloxamer-type polyether molecules are not degradable, several degradable analogs have been synthesized by exchange of the PPG block(s) with degradable hydrophobic polyester blocks, such as polylactide or poly(propylene fumarate) (PPF). The latter contains reactive double bonds along its backbone, which can be consumed in a cross-polymerization reaction [30]. Consequently, copolymers of PPF and PEG blocks, especially in the PEG-PPF-PEG composition, exhibit dual gelation properties [85,86,87]. A method for the fabrication of macroporous hydrogels has been described that made use of the excipients necessary to induce thermal cross-polymerization of the macromers. Cross-polymerization was initiated by a pair of redox initiators, ammonium persulfate and l-ascorbic acid. Through the addition of sodium bicarbonate, which reacted with the acidic component l-ascorbic acid under the formation of carbon dioxide, pores were formed and locked in the chemically cross-linked hydrogel matrix. When the copolymeric macromer were cross-co-polymerized with acryloyl-PEG-RGD, which contained the cell adhesive RGD amino acid sequence, a biomimetic hydrogel material was formed [88]. MSCs cultured on these materials were attracted to the internal pore surfaces and differentiated phenotypically to osteoblastic cells when cultured in osteogenic media.

Poloxamine-based materials are slightly different in chemistry but similar concepts have been realized to yield dual gelling macromers. Poloxamines are a family of 4-arm PPG-PEG block copolymers with an ethylene diamine core with thermo-responsive gelation properties. Following end group acrylation, the macromers can technically be cross-polymerized or cross-linked with oligovalent thiols, such as dithiothreitol, following a Michael addition mechanism [35]. Poloxamine-based hydrogels showed significantly reduced swelling and increased tensile and tissue bonding properties as compared to a PEG-based control hydrogel. This ability to maintain stable volume during equilibration may reduce the risk of compression of surrounding tissue when used as a tissue sealant.

#### 2.1.3. Poly(organophosphazene) (POP)-Based Dual-Gelling Macromers

Poly(organophosphazenes) (POP) are the third group of thermo-responsive materials that can be adjusted to form physical hydrogels at physiological temperature that is discussed here. Functional groups that have been incorporated in POP-based macromers, which are generally biodegradable, include thiol groups for cross-linking with divinyl components under physiological conditions [89]. The system was developed further towards a self-cross-linkable formulation, which consisted of a blend of thiolated and acrylated POP-based macromers. The polymer blend was soluble in physiological media at low temperature and transformed into a hydrogel with adjustable mechanical properties at body temperature without the use of monomeric cross-linkers, catalysts, oxidants, pH adjustment, initiators, UV light, heat production, or organic solvent [90].

The chemical integration of citronellol esterified amino acid units to a POP macromer introduced two additional functionalities [91]. First, the bioerosion rates could be controlled by the steric hindrance of the amino acid side chain. Second, the double bond in the citronellol structure also allowed for macromer polymer cross-linking by UV radiation (Figure 1), which aids in adjusting the mechanical properties. In addition, citronellol is an anti-inflammatory molecule. It remains to be elucidated if this property has a biological effect when the molecule is attached to the macromer or released after degradation.

## 3. Oligo-Functional Macromers with at Least One Type of Functionality not Involved in Cross-Linking

### 3.1. Thermogelling Macromers with at Least One Type of Functionality not Involved in Cross-Linking

NiPAAm-based macromers comprising other than chemically cross-linkable moieties have also been developed. Examples include thermo-responsive polyNiPAAm-*block*-poly(acrylic acid) grafted with *O*-phosphoryl ethanolamine-*block*-polyNiPAAm triblock copolymers that were synthesized by atom-transfer radical polymerization and subsequent modification [92]. Free standing hydrogels were formed from these macromers at 37 °C even at a concentration as low as 2% in PBS. Due to the pendant phosphate groups the gels induced *in situ* mineralization and hydroxyapatite formation in simulated body fluid. The material also exhibited low cytotoxicity. Materials with pendant phosphate groups have been discussed to aid in the formation of native tissue by mimicking the native role of phosphate groups in the body and by attachment of other bioactive molecules [93].

With the objective to develop a NiPAAm-based injectable hydrogel with tunable gelation, mechanical and biodegradation properties, poly(NiPAAm-*co*-(HEMAPLA)-*co*-oligo(ethylene glycol) monomethyl ether methacrylate-*co*-NAS) macromers were synthesized and conjugated with elastin via the reactive succinimide esters [94]. Adjustment of the composition of the macromer controlled degradation rate, hydrogel mechanics and gelation properties of the injectable formulations. The materials were cytocompatible and supported the growth of encapsulated dermal fibroblasts.

Injectable, thermoresponsive hydrogels composed of macromers containing NiPPAm, AAc and HEMA-oligomer (with biodegradable oligolactide, oligohydroxybutyrate, or oligo(trimethylene carbonate)) have been used to deliver stem cells into ischemic limb for enhanced myogenic differentiation and muscle regeneration [95]. As an interesting feature of the macromer, the initial hydrogel modulus could be independently varied by adjusting the molecular weight of the biodegradable oligomeric segments. MSCs were encapsulated in gels with elastic expansion moduli of 11, 20, and 40 kPa. On gels with a modulus of 20 kPa, significant myogenic differentiation was achieved after 2 weeks.

The Ameer group developed poly(NiPAAm-*co*-PEG citrate) macromers that formed thermogels with intrinsic antioxidant properties [96]. Due to the citrate groups, the hydrogels can scavenge free radicals, chelate metal ions, and inhibit lipid peroxidation. The gels have also been shown to slowly release the chemokine SDF-1α and supported the viability and proliferation of vascular cells.

NiPAAm-based macromers containing pendant RGD motifs have been developed for cell encapsulation [97,98]. Poly(NiPAAm-*co-*PEG) was synthesized from NIPAAm and succinyl PEG acrylate by free radical polymerization in benzene. The product was isolated and purified by dialysis against distilled water. The RGD grafting was performed from poly(NiPAAm-*co-*PEG) after activation with 1-ethyl-3-(3-dimethylaminopropyl)carbodiimid (EDC), dialysis and addition of GRGDS. Poly(NiPAAm-*co*-PEG-*graft*-GRGDS) was isolated after another dialysis step and subsequent lyophilization.

A dual physically gelling macromer was developed based on polyNiPAAm [99]. PolyNiPAAm end-functionalized with azobenzene groups (functionalities: thermo-responsive and ligand for intrusion complexes with cyclodextrin) was cross-linked with disulfide-connected cyclodextrin dimers to yield triple stimulus-responsive hydrogels. The sol-gel phase transition of the obtained gels was responsive to temperature, light, and reductive media.

### 3.2. Chemically Cross-Linkable Macromers with at Least One Type of Functionality not Involved in Cross-Linking

Most of the macromers discussed in this section are based on poly(ethylene glycol) (PEG). PEG-based hydrogels have progressed research on fully synthetic artificial extracellular matrices and biomimetic material design and therefore are highly popular in biomedical engineering [100,101]. In many popular approaches the macromers used to build up the hydrogels are oligovalent but each macromer typically contains only one type of chemical functionality, such as in 4- or 6-armed PEG macromers terminated with either maleimide, vinylsulfone or acrylate groups, all building blocks that can be cross-linked by Michael addition reactions [2,102]. Other effective click conjugation reactions, such as Diels-Alder or azide-alkyne cycloaddition reactions have also been established for cross-linking of PEG-based hydrogels [45,103,104,105]. According to the scope of this review, such macromers will not be discussed here because they do not contain at least two chemically different functional groups per molecule. However, some examples can be found in which researchers build up multi-functional PEG-based macromers for the design of PEG-based hydrogels. In addition, these hydrogels often comprise peptide-components in order to mediate enzymatic degradability and/or specific interactions with cells. These peptides often contain additional chemical functionalities, which qualifies them as multifunctional macromers in the sense of this review (cp. Section 4). Thus, these two types of macromers are discussed here in representation of the large variety of multi-functional PEG-based hydrogel materials developed thus far.

#### 3.2.1. Chemically Cross-Linkable Macromers with More Than One Functionality for Cross-Linking and/or Bioconjugation

Microribbons of PEG-based hydrogels have recently been introduced as interesting building blocks for the construction of 3-dimensional cell niches with independently tunable biochemical, mechanical, and topographical properties [102]. These PEG-based materials were build up from 8-armed PEG macromers and two different macromers were presented that contained different combinations of two types of chemically different functional groups in a macromer, namely PEG-(*N*-hydroxysuccinimide(NHS))_4_-(methacrylate(MA))_4_ and PEG-(NHS)_4_-(maleimide)_4_. While the latter was introduced to immobilize biochemical cues, such as RGD-peptides, via a thiol-maleimide addition reaction, PEG-(NHS)_4_-(MA)_4_ was used to adjust the stiffness of the microribbons. These macromers were synthesized from commercially available tripentaerythritol functionalized by eight poly(ethylene glycol) succinimidyl succinamide groups (PEG-(NHS)_8_).

The Elisseeff group is known for their work with PEG-based hydrogels in the context of stem cell-based regenerative applications [106]. They have presented a set of PEG-based macromers that can be derivatized and equipped with additional functional moieties via α-cyclodextrin (α-CD) nanobeads that have been threaded onto linear acrylate end-functionalized PEG molecule [107]. The cross-linking density of PEG predominantly controlled the stiffness of photo-cross-linked α-CD-PEG hydrogels independent of α-CD. Alpha-CD could be substituted with cell adhesion peptides before threading and hydrogel formation. The concentration of cell integrin-binding peptide conjugated to α-CD could be varied independent of the cross-linking density. Other chemical modification of α-CD were also described (*i.e.*, hydrophobic, hydrophilic or charged groups) for the engineering of specific microenvironments. The spatially dynamic presentation of biochemical cues with this system is a unique feature.

#### 3.2.2. Chemically Cross-Linkable Macromers with Cell Adhesive Motifs

Third-generation biomaterials are materials that are biocompatible, resorbable and bioactive [108,109]. Target values of these parameters for the design of such materials greatly depend on the biological system, tissue or type of cells the materials should interact with in the desired application. Especially the property “bioactivity” is defined by many physico-chemical parameters of the material; these include bulk mechanical properties, the extent of protein absorption to the material, and the ability of the material to directly interact with adhesion-mediating proteins on the surface of cells. This motivated research towards the development of injectable hydrogels that readily integrate with specific tissues or specifically interact with invading or encapsulated cells. Cell adhesion promoting peptide sequences, especially those containing the tripeptide sequence RGD, play a crucial role in such strategies [110,111]. In early strategies, material surfaces have been coated with cell adhesive proteins from ECM such as collagen, fibronectin or laminin. Such protein coatings were associated with many disadvantages mainly host infection risk, immunological reactions, little specificity, and inadequate stability owing to higher proteolytic degradation. Many of these problems can be resolved by substituting the protein molecules for short immobilized motifs. Such motifs have shown many advantages over large protein molecules including stability to sterilization conditions, dense packing on surfaces because of their small size, high specificity, less enzymatic degradation and good stability. The RGD motif is present on many ECM proteins, e.g., fibronectin, collagen and laminin, bone sialoprotein, plasma membrane, and in many bacterial and viral proteins. RGD peptides facilitate all overlapping phases of cell adhesion *i.e.*, attachment, spreading, association of actin cytoskeleton, and focal adhesion formation [112].

Strong cell adhesion requires a firm attachment of RGD to a substrate. Therefore, motifs should be covalently bound to polymers through functional groups like amino-, hydroxyl or carboxylic groups [110]. A common strategy to introduce RGD-type peptides in synthetic hydrogels, is the use of heterobifunctional linkers, such as acryloyl-PEG-RGD, during a chemical cross-copolymerization reaction with other hydrogel-building macromers. Such heterobifunctional molecules technically meet the criteria for macromers that are discussed in this review. However, due to the high number of similar structures available in the literature and the commercial availability of some of these linkers, we will discuss only a few selected examples. One example as already given above, where biomimetic PEG-PPF triblock copolymer-based networks were described [88]. Another study cross-copolymerized acryloyl-PEG-RGDS and photo-cross-linkable azidobenzoic acid-modified chitosan. C2C12 myoblasts were mixed with aqueous solutions of both macromers and the dispersion was cross-polymerized by UV irradiation (4 mW/cm^2^) [113]. The hydrogel was discussed for the delivery of cells and growth factors to injured myocardium. When a hetero-bifunctional PEG-based macromer contains photodegradable *ortho*-nitrobenzyl (cp. Section 3.3.2), the RGD peptide can be released on demand, which can be beneficial for the generation of gradients [114].

Chemically cross-linkable RGD-functionalized PEG macromers were synthesized from 4-arm PEG macromers (PEG-tetra acrylate, PEG-tetra maleimide, PEG-tetra vinylsulfone) that were reacted with a thiol-containing adhesive peptide GRGDSPC in PBS at pH 7.4 under stoichiometric control [115]. The mono RGD-functionalized PEG macromers were subsequently cross-linked into a hydrogel by the addition of a protease-cleavable peptide cross-linker GCRDVPMS↓MRGGDRCG that contains thiol groups in cysteine (C) side chains [116]. The degradable PEG-protein pentamer-based hydrogel described earlier [117] contains RGD motifs within one of the genetically engineered protein pentamers in order to mediate specific interactions with cells.

Further examples for RGD-peptide-containing hydrogels are illustrated in Section 4. In all examples discussed there a peptide-based macromer is the key building that has at least dual functionality in the sense of this review.

#### 3.2.3. Chemically Cross-Linkable Macromers with Other Additional Properties

Motivated by the natural association of bone apatite crystals with citrate-rich molecules, a citrate-based injectable composite material for orthopedic applications was developed [32]. A PEGMAC macromer was synthesized by a simple and controlled polycondensation reaction of PEG, maleic anhydride (MA) and citric acid (C). PEGMAC can be cross-copolymerized, as done with PEG-diacrylate in this study, via the double bond of maleic anhydride and strongly interact with hydroxyapatite (HA) via citrate molecule. Cross-linked PEGMAC/HA composite is considered a promising injectable cell carrier for orthopedic regeneration.

### 3.3. Macromers with Advanced Degradative Properties

Hydrogels are favorable matrices for drug and cell delivery due to their high water content and mechanical properties similar to natural tissues [118]. The applicability of a hydrogel material for such applications, however, is critically dependent on the degradative properties of the material. Bioerosion, *i.e.*, loss of hydrogel mass over time in contact with a biological system, of hydrogel materials, either of natural or synthetic origin, can be the consequence of unspecific aqueous hydrolysis or enzymatic degradation of chemical bonds in the material and the formation of soluble fragments at physiological conditions. Different degradation mechanisms are known depending on the chemical nature of material and cross-links [119,120]. In physically assembled hydrogels, slow dissolution of the constituent amphiphilic molecules is often the primary degradation mechanism, even when slowly degradable amphiphilic polyesters were used [121]. In order to increase the solubility of degradation fragments or complete macromer building blocks, hydrolytic reactions can be used to increase the hydrophilicity of pendant groups. This strategy can be particularly helpful, to increase the solubility of thermally gelled macromers at physiological temperature [60]. The third, generally different, mechanism involves the chemical degradation of bonds along the backbone of the constituent macromers and/or cross-linkers within the cross-linked material [4]. This mechanism can include: (1) the unspecific hydrolysis of carboxylic acid ester, carbonate esters, carbamate ester, or anhydride bonds, among others; (2) the reductive, intracellular degradation of disulfide bonds; (3) the enzymatic degradation of specific peptide motifs; (4) other stimulus-induced degradation of specific chemical bonds, such as photodegradation and reversible click reactions. Examples of dual-functional macromers that contain hydrolytically labile ester groups along the backbone have already been presented in this review [87,122] and will not further be detailed.

#### 3.3.1. Oligofunctional Macromers Sensitive to Reductive or Enzymatic Degradation

PEG-based macromers have been made biodegradable through the integration of phosphate ester groups [123]. PEG-di-[ethylphosphatidyl(ethylene glycol) methacrylate] has been synthesized from anhydrous PEG, ethyl dichlorophosphate and HEMA. The water-soluble macromer was suitable for *in situ* injection and cell-encapsulation by light-induced gelation. Human MSCs (hMSCs) were encapsulated and remained viable. Another phosphate-containing, biodegradable macromer is poly(6-aminohexyl propylene phosphate) that was photo-cross-linked into elastic hydrogels [124]. Goat MSCs were encapsulated and could be differentiated to deposit mineralized extracellular matrix.

Thermogelling poloxamer-disulfide macromers are an example for dual-functional, reductively degradable macromers [125]. Hydrogels from disulfide cross-linked poloxamer remained stable in buffer, while uncross-linked gels disintegrated within several hours. The cross-linked gels eroded and released an encapsulated model drug in a glutathione concentration-sensitive manner.

Examples for oligo-functional peptides are discussed in Section 4. An enzymatically degradable PEG-based, chemically cross-linkable macromer has been described by Miller and coworkers [126]. Macromers with the general structure acrylate–PEG–(peptide–PEG)_m_–acrylate were obtained by step growth polymerization of PEG-diacrylate and bis-cysteine peptide sequences. Three different sequences were utilized with different susceptibility to degradation by matrix metalloproteinases (MMPs). In a similar approach, degradable protein pentamers were incorporated in PEG-based macromers by Michael addition chemistry [117]. Two artificial pentamers were prepared by recombinant DNA technology using human gene codes. One pentamer contained functional RGD and plasmin degradable sites, while the other comprised non-functional RGD and plasmin non-degradable site with almost 10 point mutations. The pentamers were modified with acrylated PEG molecules that were conjugated with thiol groups of the pentamers. The macromers contained an access of acrylate groups that enabled hydrogel formation by photopolymerization. The resulting gels were cell adhesive, degradable by tissue enzymes, had suitable mechanical strength and showed cellular movement for almost 9 days.

#### 3.3.2. Photodegradable Oligofunctional Macromers

A chemical moiety that imparts photodegradability to a macromer is 4-(4-(1-hydroxyethyl)-2-methoxy-5-nitrophenoxy)butanoic acid (or: *ortho*-nitrobenzyl (*o*-NB)) (Figure 5a). The *o*-NB moiety contains both a carboxylic acid and a benzylic alcohol, allowing for separate functionalization of these two moieties. A library of polymerizable PEG-based *o*-NB-containing macromers with different functionalities at the benzylic position (alcohol, amine, BOC-amine, halide, acrylate, carboxylic acid, activated disulfide, *N*-hydroxysuccinyl ester, biotin) have been synthesized for hydrogel formation, cell encapsulation and conjugation of therapeutic agent, such as cell adhesive peptides, cytokines or growth factors [114,127]. The photodegradable moieties offer precise external temporal and spatial control over network erosion and/or drug release. A controlled photorelease of growth factors and cytokines can be used to generate gradients and guide cell homing, growth and migration and ultimately tissue development. As light energy is a parameter that can be applied externally with precise control, e.g., by two-photon confocal excitation, such macromers hold promise for use in highly complex tissues engineering.

A hydrogel system based on two types of dual-functional macromers, namely a maleimide end-functionalized, photodegradable macromer containing an *o*-NB ether (functionalities: maleimide and *o*-NB) and a four-arm PEG end-functionalized with aryl thiols (functionalities: hydrolytically labile ester and thiol groups), has been described that combined three different cleavage chemistries in order to engineer multimodal degradable hydrogels for responsive and triggerable modulation of gel properties and cargo release [128]. The injectable hydrogel formed *in situ* by a Michael addition reaction between thiols and photodegradable maleimides. The resulting succinimide thioether linkages were sensitive to thiols and reducing environments. Consequently, this specific degradation mechanism (of the succinimide thioether) qualified as cleavage of click linkages under physiological conditions [129]. The *o*-NB-containing macromer provided photodegradability and ester linkages in the macromers were sensitive to hydrolysis. The hydrogels could be degraded in a precisely controlled manner and degradation mechanism could be switched between surface and bulk degradation.

Coumarin has recently been introduced as a potential alternative to the previously described *o*-NB-based systems [130]. A particular advantage of coumarin as photodegradable group is a high biocompatibility as the photodegradation products are biologically inert in contrast to the reactive carbonyls that are released upon *o*-NB degradation. In addition, coumarin can be degraded by a broader spectrum of cytocompatible wavelengths of light. An amine-terminated coumarin azide was synthesized and conjugated to a 4-armed PEG yielding the dual-functional 4-armed PEG tetra-coumarin-azide. Via an aqueous copper-catalyzed click reaction with 4-armed PEG tetra-alkyne coumarin-based photodegradable hydrogels were obtained. Degradation experiments revealed that the degradation kinetics at 405 nm were approximately 10 times slower than at 365 nm.

### 3.4. Hydrogel-Forming Macromers with Shape Memory Properties

Shape memory is a property of selected materials that can recover a predefined shape from an interim, deformed pseudo-plastic state in response to a change in environmental conditions [131,132,133]. Shape memory materials, especially polymers and hydrogels, are therefore considered as stimulus-responsive materials. Shape memory materials have been developed that respond to different triggers, but temperature change remains the most investigated stimulus. Shape memory mechanisms can be significantly different depending on the type of material [133,134]. This property cannot be related to one specific functional group or structural component, but rather depends on the specific combination of structural components and physico-chemical parameters. Advantages that are attributed to shape memory materials including hydrogels are that their surgical application requires minimal invasion, that they can be used for one time or cyclic tissue actuation and that patterned surfaces for controlled cell adhesion and growth can be generated [133]. Typical shape memory hydrogels (SMGs) are chemically cross-linked materials that contain hydrophilic domains that swell in water and hydrophobic domains that are poorly hydrated and stimulus-responsive. Biodegradable SMGs are usually preferred in order to avoid the need of a second surgical intervention to remove the implanted device. Thermo-responsive SMGs normally have their original shape at high temperature where molecular net-points are linked. In response to a decrease in surrounding temperature, hydrophobic moieties tend to establish physical cross-links and maintain a temporary deformed shape. At this temperature, molecular segmental motion is impossible and SMGs lose the ability to restore their original shape. An increase in temperature can provide the energy for molecular segmental motion to enable recovery of the original shape. Several examples of SMG are cross-linked from monomer mixtures [134,135]. We found only one example so far that represented a macromer-based SMGs [136]. The gel was based on amphiphilic macromers with terminal acrylate groups for chemical cross-linking. The macromers further consisted of hydrophilic PEG blocks and hydrophobic poly-1,4-butylene adipate glycol and 3-isocyanatomethyl-3,5,5-trimethylcyclohexyl isocyanate. The amphiphilic macromers formed micelles with a hydrophobic core, which was stabilized by hydrogen bonds within the hydrophobic domains of the macromers. The copolymerized, equilibrium swollen micellar hydrogel showed high ductility and high resilience. Beside these properties, the networks exhibited water-responsive shape-memory properties because of a dehydration-induced glass transition and a plasticizing effect of absorbed water molecules.

## 4. Peptide-Based Oligo-Functional Macromers

Synthetic peptides have become key components in the design of hydrogels that mimic ECM as they can impart cell adhesive properties and enzymatic degradability to the final gel [4,100]. Versatile material platforms can be designed through the combination of peptidic macromers and biologically inert polymeric macromers (Table 1). Enzymatic degradation, for example by matrix metalloproteinases (MMPs), facilitates cell cultivation, differentiation, and migration as well as matrix remodeling.

**Table 1:** Combinations of functional properties realized in peptide-based macromers as discussed in this review. *cCL* stands for chemical cross-linking and *conj.* illustrates that the corresponding functional groups allow for cross-linking via conjugation chemistry.

Properties	Photo-Degradability	cCL: Radical Polymerization	cCL—conj.: Azide—Alkyne	cCL—conj: Diels-Alder	cCL—conj.: Thiol-Ene, Michael Addition
Enzyme degradability	[137]	[117]	[104,137]	[138]	[104,139]
Cell adhesive domains	-	[117]	[137]	[138]	-
cCL—conj: Diels-Alder	-	-	-	-	[138]

**Figure 6:**
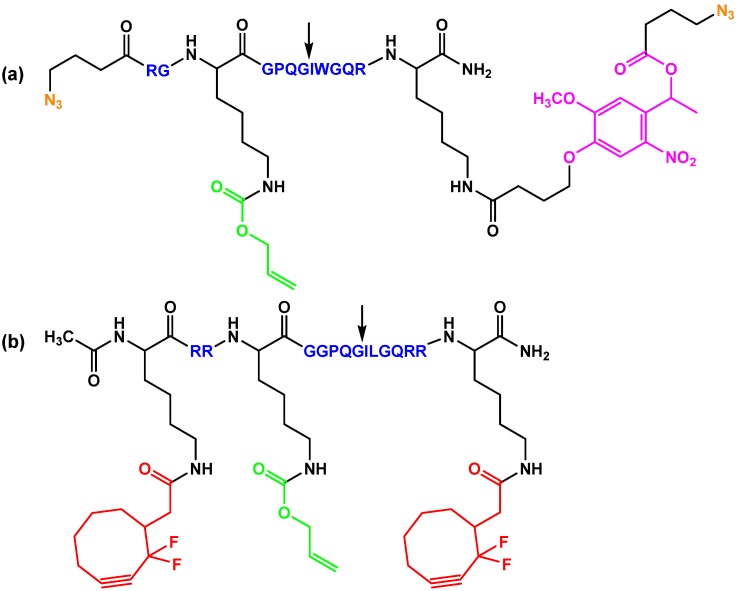
Enzymatically degradable peptide-based macromers with different combinations of functional moieties. An arrow indicates the enzymatic degradation site in the peptide sequence. (**a**) Peptide with photodegradable *o*-NP ester (magenta) and functional moieties for thiol-ene addition reactions (green) and azide-alkyne cycloaddition chemistry (orange) [137]; (**b**) Peptide with functional moieties for thiol-ene addition reactions (green) and strain-promoted azide-alkyne cycloaddition chemistry (red) [104].

The Anseth group, for example, developed a well-based cell culture platform that allowed for the spatiotemporal control of geometry and connectivity of cellular microenvironments on the basis of a PEG-based hydrogel that contained a multi-functional peptide macromer [137] (Figure 6a). The peptide N_3_-RGK(alloc)GPQG↓IWGQRK(PL-ester-N_3_)-NH_2_ is photo-labile, enzymatically degradable (for example by MMPs 1, 2, 3, 8, and 9; arrow indicates site of cleavage) and functionalized with azides. The peptide also contained an allyloxycarbonyl (alloc) group that can be conjugated with a thiol group-containing bioactive molecule in a photo-catalyzed thiol-ene reaction. These functional groups allow for copper-free click hydrogel formation and patterning or cell-driven enzymatic remodeling in combination with orthogonal light-based degradation. RGD sequences were integrated as azide-RGDS or bis-azide-RGDS. The robust click reaction resulted in instantaneous gelation that was completed in 1 h.

Epithelial cells were cultured in the hydrogel wells in defined geometries to demonstrate the utility of this platform, which can be helpful to understand how micro-environmental cues influence cell cluster shapes, cell–cell interactions, and ultimately cell function and fate during tissue development or regeneration. In a similar example, a MMP-degradable peptide sequence (GPQG↓ILGQ) represented the degradable domain in a peptide macromer of the following structure: Ac-KRRK(alloc)GGPQGILGQRRK–NH_2_ [104]. In addition, difluorinated cyclooctyne (DIFO3) was coupled to the ε-amino groups of the terminal lysines incorporating a strain- and fluorine-activated alkyne for copper-free azide-alkyne cycloaddition reactions (Figure 6b). For hydrogel formation, the peptide macromer was cross-linked with four-arm PEG tetra-azide. As intended by the hydrogel design, the enzyme degradable sequence permitted better cell viability and migration as compared to gels prepared without such sequences. In addition, the functionalities that have further been attached to the peptide allowed these macromers to become central building blocks that control hydrogel properties. The same cross-linking strategy was used to build up hydrogels from four-arm PEG tetracyclooctyne and a functionalized polypeptide N_3_-RGK(alloc)GRK(PL-ester-N_3_)-NH_2_ [140]. Via a thiol-ene coupling to the *alloc* group, cell adhesive thiol-containing RGD sequences were incorporated to improve cell spreading and migration in the gels.

Another, effective “click” conjugation reaction, that has been used for the chemical assembly of PEG-peptide-based hydrogels is an IEDDA reaction [138]. A dinorbornene (norb)-functionalized cell degradable peptide, norb-KGPQG↓IWGQKK-norb, was reacted with PEG-benzylaminotetrazine in stoichiometric quantities with the release of nitrogen gas as by product (Figure 2). Norbornene-modified RGD motifs (norb-AhxRGDS) were also incorporated to control hydrogel interactions with cells. The system presented an excellent environment for encapsulated hMSCs with easy and accessible chemistry.

Michael-type addition reactions, another efficient but not biorthogonal bioconjugation technique were also used for reactions between peptide and PEG macromers. A peptide sequence containing a MMP-sensitive and a cell adhesive domain (NH_2_-GGRGDGPQG↓IWGQGGCG-CONH_2_) was conjugated to 4-armed PEG tetraacrylate via the thiol group of cysteine in the peptide [139]. The peptide-PEG-macromer presented free amino groups in the attached peptides that reacted via carbodiimide chemistry to chemically modified heparin using carbodiimide/sulfo-NHS and led to gelation in 1 hr. The system allowed for the encapsulation of viable cells and took advantage of enhanced binding properties of heparin for many chemotactic factors for delivery applications. The platform of PEG/GAG networks has been expanded for different biomedical applications [141,142].

Similar chemistry has been utilized to form a network between double bond containing PEG molecules derivatized with acrylate, vinylsulfone or maleimide and thiol-functionalized RGD or MMP sequences, for example dithiol protease-cleavable peptide cross-linker GCRDVPMS↓MRGGDRCG [115]. Hydrogels were formed by the addition of predefined volumes of acrylate-PEG-RGD-MMP-acrylate solutions and photoinitiator to the peptide solution and subsequent exposure to UV light. PEG-tetramaleimide cross-linked hydrogels presented better properties as compared to other composition as the mechanical properties of these gels were comparable to ECM like collagen type I. Furthermore, the gels formed quickly (less than 5 min) and encapsulated cells showed increased spreading.

## 5. Macromers Based on ECM-Molecules or Biologically Active non-ECM Polysaccharides

In the context of this review, many ECM components or biologically active non-ECM polysaccharides that are derivatized chemically and are capable of forming hydrogels qualify to be included as they may contain two or more different functional properties. The first functionality is the inherent bioactivity of the ECM molecule or polysaccharide. The second functionality can be manifold and different structures have already been described in which the second functionality contributes to increased hydrogel stability as it allows for physical or chemical cross-linking. With regard to physical cross-linking, thermoresponsive macromolecules have been combined with modified or basic ECM components or non-ECM polysaccharides. Sensitivity to environmental temperature may be implemented by cellulose derivatives [143,144], poloxamer [145], or the extensively used thermoresponsive polyNiPAAm [94,144,145,146,147,148,149,150,151]. A variety of natural macromolecules have been modified, including hyaluronic acid [147,148], deacetylated chitosan [145], cellulose derivatives [143,144], laminin [143], elastin [94], hydrazide-functionalized chondroitin sulfate [146], gelatin [149,151,152], and maleic anhydride-coupled dextran [150]. A variety of chemistries has been employed to conjugate or graft-polymerize thermo-responsive moieties to these molecules. Examples include graft polymerization after oxidative ring opening of polysaccharides [143,145] or conjugation of a reactive double bond [150], atom transfer radical polymerization from bromoisobutyryl-functionalized cellulose [144], RAFT polymerization and condensation [146], dithiocarbamyl iniferter (initiator, transfer agent and terminator)-based photopolymerization [151,153], and amide bond formation [145,148].

### 5.1. Hyaluronic Acid (HA)-Based Macromers

HA is a linear high molecular weight acidic ECM polysaccharide of alternating d-glucuronic acid and *N*-acetyl-d-glucosamine. HA offers a broad spectrum of biomedical properties as it participates in many biological processes including cell proliferation and differentiation, morphogenesis, inflammation, and wound healing [154,155,156]. HA can interact with cells and signaling molecules through specific and non-specific interactions. HA is a ligand to certain receptors and cell surface proteins, most prominently CD44, and can thereby affect cellular signaling pathways [157]. HA is also involved in inflammatory processes, wound healing and scar formation. In these processes, HA molecular weight plays a decisive role. Generally, high molecular weight HA is considered to favor cell quiescence in these processes and supports tissue integrity, whereas HA fragments act as so-called danger signals that indicate cell or tissue damage and initiate an inflammatory response. In view of these properties, HA-based hydrogel systems are popular matrices in regenerative applications focusing on wound healing, cartilage defects and joint disorders, osteoarthritis, valvular engineering and cardiac repair [158]. In order to obtain the required hydrogel stability or to adjust the mechanical properties of the gels to a defined application the introduction of chemical moieties that enable physical or chemical cross-linking of HA molecules is often necessary. HA can be conveniently functionalized at its carboxylic and hydroxyl groups as well as at amine groups that can be made available by deacetylation. Typical modifications of the carboxylate have been described by carbodiimide-mediated esterification, amidation or hydrazidation. The hydroxyl groups have been predominantly derivatized by etherification and esterification to introduce additional functionalities. In order to enable physical gelation, polyNiPAAm that has been introduced by graft polymerization in one example, which resulted in a water soluble macromer that underwent lower critical phase separation upon heating above 34 °C [153]. HA-based macromers with chemically cross-linkable moieties allowed for the fabrication of gels with significantly increased stability and for the application of biofabrication techniques. Typical examples include HA-methacrylate, HA-hydroxyethylacrylate, HA-tyramide, HA-hydrazide, and thiol-modified HA [158,159]. Cross-linkable HA has been commercialized as a semi-synthetic extracellular matrix, HyStem^®^ (ESI BIO, Alameda, CA, USA) [160]. The composition of this material can be customized for use with adult progenitor and tissue-derived cells, MSCs as well as cells obtained from embryonic or induced pluripotent sources.

An interesting HA macromer was described that formed injectable, temperature-responsive, covalent networks with thiol end-capped poloxamer [161]. Inspired by functional domains of mussel adhesion proteins, HA was conjugated with dopamine using carbodiimide chemistry. The macromer was mixed with poloxamer F127-dithiol to produce a lightly cross-linked composite gel by Michael-type catechol-thiol addition reaction. The gel could be injected in a sol state at room temperature using a syringe and immediately became a robust gel state at body temperature. The *in situ* formed hydrogels exhibited excellent tissue-adhesion properties.

An HA-based macromer with two types of functional groups in addition to the inherent biological functionality of HA has been described by the Burdick group [162]. Through the conversion of a tetrabutylammonium salt of HA with succinylated hydroxyl ethyl methacrylate in a catalyzed esterification reaction, chemically cross-linkable HEMA-HA was obtained that, in a second reaction, was chemically sulfated by the addition of SO_3_/DMF complex in DMF to obtain sulfated HEMA-HA. This macromer could be covalently incorporated in chemically cross-linked HA gels and the formulations was processable by electrospinning to yield cross-linked hydrogel nanofibers. The sulfate groups enable controlled interactions with heparin-binding growth factors and cytokines, here shown for SDF-1, and sustained the release of such biological molecules.

### 5.2. Gelatin-Based Macromers

Gelatin is a fraction of polypeptides that are obtained by partial hydrolytic degradation of collagen and contains RGD sequences that can directly interact with integrins on cell surfaces and promote cell adhesion [120]. The peptides also contain matrix metalloproteinase-sensitive degradation sequences. Gelatin is inexpensive and compared to collagen it has superior water solubility, processability and lower immunogenicity. The inherent bioactivity, as well as biodegradability of gelatin, makes this polypeptide an attractive precursor for the fabrication of cell-laden hydrogels [57]. Thermogelling gelatin macromers have been obtained by grafting polyNiPAAm to dithiocarbamylated (iniferter-derivatized) gelatin in a controlled photo-initiated polymerization reaction [149,152,163]. Chemically cross-polymerizable gelatin can be obtained by methacrylation [164]. Gelatin macromers that allow for bioorthogonal click conjugation have also been described [165]. Dibenzylcyclooctyne-C4-acid (DBCO)-modified gelatin and photodegradable azide-*o*-NB-modified gelatin as well as PEG-tetra-DBCO and PEG-tetra-*o*-NB-azide were synthesized. Strain-promoted azide- alkyne cycloaddition between azide and DBCO moieties yielded cross-linked, fully photodegradable gelatin-PEG hydrogels. The photodegradation kinetics was rapid, and the gels were completely degraded within 2−3 min upon irradiation with UV light. Fibroblasts were encapsulated within the gels and demonstrated high cell viability after 2 weeks. Encapsulated cells could be released on-demand and with high viability through UV irradiation after 1, 3, or 7 days.

### 5.3. Gellan Gum (GG)-Based Macromers

GG is a water-soluble, anionic bacterial polysaccharide secreted by the bacterium *Sphingomonas elodea* with repeating tetrasaccharide units of d-glucose, d-glucuronic acid, d-glucose, and l-rhamnose [166]. The biocompatibility of the material has been shown for different biomedical applications [167]. Gellan gum is a thermoresponsive material with a sol-gel transition upon cooling and can be ionically cross-linked [168]. Upon methacrylation with methacrylic anhydride or glycidyl methacrylate, gellan gum derivatives were obtained that could be gelled by combinations of physical (temperature and the addition of cations) and chemical (photo-cross-linking) cross-linking methods [169,170]. Physical and mechanical properties of the resulting gels could be highly tuned without affecting their biocompatibility. Cross-linked gellan gum gels have been proposed for the challenging application as acellular or cellular substitutes of the nucleus pulposus [170]. The hydrogels were mechanically compliant with this application, did not elicit any acute inflammatory response and functioned as a physical barrier for vascular invasion [171]. Human bone marrow-derived MSCs and nasal chondrocytes (NCs) were encapsulated in both ionically and chemically gelled gellan gum hydrogels and remained viable for at least 2 weeks without signs of *in vivo* chondrogenesis [172].

### 5.4. Chitosan-Based Macromers

Chitosan is a non-physiologic glycosaminoglycan with interesting properties. It has been described to exhibit good biocompatible and antimicrobial properties [173,174]. Chitosan-based materials have been used to accelerate wound healing and to support the proliferation and differentiation of osteoblasts and chondrocytes. On the flipside, chitosan has unfavorable solubility properties. In order to overcome this limitation and to incorporate chemically cross-linkable domains, chitosan was derivatized into glycol chitosan and made chemically cross-linkable by subsequent methacrylation using glycidyl methacrylate in aqueous solution [175]. The macromer possessed the same non-cytotoxicity as non-methacrylated glycol chitosan toward an immortalized chondrocyte cell line. Photo-cross-linked gels were enzymatically degradable and supported the growth of immortalized chondrocytes over a period of one week.

## 6. Conclusions

A large variety of interesting hydrogel-forming macromers has been developed over the last decades (Table 1 and Table 2). Among those, this review focused on macromers with at least two chemically different functionalities, including functional groups or domains for physical or chemical cross-linking, bioconjugation, advanced degradative properties, and defined interactions with cells. The macromers we identified were fully synthetic polymeric molecules, chemically derivatized peptides and derivatives of extracellular matrix components and natural polysaccharides. Peptide-based macromers have been synthesized with combinations of functional groups for effective click conjugation chemistries thus far not realized in synthetic oligo- or polymeric macromers possibly because of the opportunities for functional group incorporation provided by state-of-the-art peptide synthesis techniques. Biomaterial scientists will continue to develop multi-functional hydrogel building blocks and additional combinations of functional domains to those discussed here will be realized in macromer design providing novel building blocks for tailored hydrogel engineering for regenerative applications.

**Table 2:** Combinations of functional properties realized in macromers discussed in this review. *pCL* abbreviates physical cross-linking, *cCL* stands for chemical cross-linking and *conj.* illustrates that the corresponding functional groups allow for cross-linking via conjugation chemistry.

Properties		Specific Degradability	Cell Adhesive Domains	pCL: Hydrogen Bonding	pCL: Ionic Cross-Linking	cCL: Radical Polymerization	cCL—conj.: Azide-Alkyne	cCL—conj: Diels-Alder	cCL—conj.: Thiol-ene, Michael Addition	cCL—conj.: Amide/Amine Formation	cCL—Other Mechanism	Other
pCL: stimulus-responsive, hydrophobic effect	NiPAAm-based, other acrylamides	-	[97]	-	-	[52,53,62]	[70]	[68,69]	[36,42]	[56,57,58,59,60,64,94]	[50,74]	[50,55,56,62,92,94,95,96,99]
-	block copolymers	[125]	-	-	-	[78,79,80,81,85,86,87]	-	-	[35,83]	-	[82]	-
	Poly (organophos-phazene)s (POP)	-	-	-	-	[91]	-	-	[89,90]	-	-	[91]
cCL: radical polymerization		[126]	-	-	-	-	-	-	[102]	-	-	[32,102,123,124]
Photo-degradability		-	-	-	-	[114,127]	[130,165]	-	[128,129]	-	-	[114]
Cell adhesive domains		[139]	-	-	-	[88,107,113]	-	-	[115,139]	-	-	[107]
ECM molecule		[165]	-	-	-	[113,122,162]	[165]	-	[161]	-	[161]	[122,161,162]
Bioactive non-ECM polysaccharide		-	-	-	[168]	[168,169,175]	-	-	-	-	-	-
Shape-memory gels		-	-	[136]	-	[136]	-	-	-	-	-	-
